# Creating engaging, safe, non-threatening, psycho-educational and FUN multi-patient/family group sessions for adults with eating disorders and their families in a day patient setting

**DOI:** 10.1186/2050-2974-2-S1-O32

**Published:** 2014-11-24

**Authors:** Liz Wynne, Lisa Stokes

**Affiliations:** 1 Eating Disorders Program, NorthWestern Mental Health, Royal Melbourne Hospital, Melbourne, Australia

## 

Over the last three years both patients, families and significant other have been actively involved in the multi-patient/family group sessions. To date 40 patients and 60 families have engaged in these groups. All sessions have been evaluated and feed-back has been positive with 100 % of both patients and families stating they would definitely recommend them to others. So what is it about these groups that works? What are the difficulties/ limitations of these groups?

This workshop will briefly outline the structure of the group sessions, present data analysis and findings. It will highlight strategies that capture and engage both patients and families and enable a trusting safe environment to be established. Lastly, it will consider if a small recovery goal or change/shift created together with the patient and family can carry through from the actual session into the "real world". Findings of this specific work will be shared.

This abstract was presented in the **Parental Roles in Prevention and Support** stream of the 2014 ANZAED Conference.

